# The influence of nutrition knowledge, attitude, practices, and dietary diversity on obesity among market women in the Cape Coast Metropolis, Ghana: A cross‐sectional study

**DOI:** 10.1002/hsr2.1742

**Published:** 2023-12-06

**Authors:** Charles Apprey, Martina Mensah, Desmond Agyarko, Yaa Asantewaa K. Klu, Samuel Acquah

**Affiliations:** ^1^ Department of Biochemistry and Biotechnology, College of Science Kwame Nkrumah University of Science and Technology Kumasi Ghana; ^2^ Faculty of Life Sciences, Food, Nutrition and Health University of Bayreuth Bayreuth Germany; ^3^ Department of Food Science University of Wisconsin‐Madison Madison Wisconsin USA; ^4^ Department of Medical Biochemistry, School of Medical Sciences University of Cape Coast Cape Coast Ghana

**Keywords:** dietary diversity, market women, nutrition knowledge, obesity, visceral fat

## Abstract

**Background and Aims:**

Obesity and overweight are major public health threat affecting many people globally. This study aimed to examine the role of nutrition knowledge (NK), attitude, practices, and dietary diversity (DD) on the prevalence of obesity among market women in Cape Coast, Ghana.

**Methods:**

This cross‐sectional study was conducted at Abura and Kotokuraba markets in the Cape Coast Metropolis of Ghana. Apparently healthy female traders (*n* = 402) aged ≥18 years were selected randomly from the markets. DD was assessed with dietary diversity score (DDS) using a 24h dietary recall method. NK, dietary practices, and attitudes were assessed using validated semistructured questionnaires. Body composition parameters were assessed using appropriate tools. Descriptive and binary logistic regression analysis were performed. Statistical significance was considered at *p* < 0.05.

**Results:**

The prevalence of overweight was 31.84% and obesity was 39.30%. Majority of respondents had poor DD as about 91% had DDS <5. About 75% of the market women had no knowledge in nutrition. About 57% eat thrice daily and 82% take supper from 7 p.m. Knowledge in nutrition was significantly associated with body fat (OR = 0.45, 95% CI = 0.26–0.78, *p* = 0.004), body mass index (OR = 0.40, 95% CI = 0.28–0.71, *p* = 0.001), and waist‐to‐hip ratio (OR = 0.32, 95% CI = 0.19–0.56, *p* < 0.001)

**Conclusion:**

The prevalence of obesity and overweight was high among the market women. Poor NK and poor DD may have influenced this. A campaign on better dietary practices and delivery of nutrition education may help to minimize the prevalence of obesity among market women.

## 1 INTRODUCTION

Obesity and overweight are significant public health threat projected to affect more than one billion adults worldwide by 2030.^1^, ^2^, ^3^ This challenge undermines social and economic development globally, rising inequalities within populations and among countries. Such high economic and social impact result in increase in poverty^4^ and reduced quality of life.^5^ In 2019, obesity contributed to about five million deaths from cardiovascular diseases, diabetes, cancers, neurological disorders, respiratory diseases, and so forth.^6^ Other publication by Oladoyinbo et al.^7^ corroborate this finding of the effect of obesity and overweight to complicate diseases and result in death.

In Ghana, this high prevalence of obesity and overweight has been reported in several publications. Asosega et al.^8^ reported that among reproductive women, the overall prevalence of overweight/obesity was 35.4%. Asenso et al.^1^ also reported nearly 43% fat or obese in adults, with 36.9% prevalence in the Central Region, and Tuoyire et al.^9^ reported the prevalence of obesity/overweight is about 39% with higher rates among women (50%) than men (28%) of Cape Coast residents. In Nigeria, Awosan et al.^10^ has reported a prevalence of 28.9% and 28.1%, respectively for overweight and obesity for market traders at Sokoto state.

In the year 2000, the population of Ghana was 18.9 million,^11^ and women constituted 50.52%. In the 2021 census,^12^ the population was 30.8 million with women representing 50.7%. As the population of the country increases over the decades, so has been the population of women, and is expected to reach beyond 51% in the next decade. This makes women the majority of the people and majority of the these women work in the informal sector.^13^ With the current census, Central Region with Cape Coast as its capital has a population of almost 2.9 million, of which women constituted 51.4%. The region is part of four regions that make up more than 54% of the entire population of Ghana, with an annual intercensal population growth rate of 2.4%, above the national average of 2.1%.

Market traders are part of the informal labor sector^14^, ^15^, ^16^ and their working environment plays a significant role in their social life.^10^ This working environment has become the “home” of these traders^16^ as they spend most of their time there. This can influence their dietary intake, nutritional status, and their overall health. Most of these traders have no means of preparing their food while at the market,^17^ and rely on food vendors who are stationed within and around the markets. These traders mostly spend all their time at the workplace^18^ and harldy get time to visit the hospital for medical check‐ups. Some of these traders habour the fear of loosing some earnings when they close their stalls or sheds, eventhough they are predisposed to obesity and overweight and its accompanying adverse health effects due to the sedentary nature of their work.

Having a good nutrition knowledge (NK) equips a person with the awareness and appreciation of concepts and processes linked to health and nutrition.^19^ Thus, an enlightened person is endowed with greater NK,^20^ and aided in improved choice of good food to prevent obesity. Another factor influencing obesity and overweight is attitude and practices of individuals with food choices^21^ and these are cultural values which can influence eating behaviors.^22^ It is common around the world that food intake be divided into three; breakfast, lunch, and super.^23^ Frequently eating (snacking) has been recommended to be a strategy for losing weight. This has been presumed to decrease hunger,^24^ energy intake, and body weight. The dietary diversity score (DDS)^25^, ^26^ is an index that defines nutrition adequacy and diet quality. DDS assesses the diversity within food groups that are frequently consumed based on a healthy dietary guidelines. Highly diversified food groups like vegetables and fruits could raise DDS without adding substantial energy to the overall calorie content of a diet, and this may result in a lower body mass index^27^ and a lower cholesterol.^28^

Halting this rising trend of obesity and overweight is a key Global Nutrition Target and one of the targets for reducing noncommunicable diseases (NCDs) in adults. If obesity is not addressed, achieving a 30% reduction in premature deaths from NCDs by 2030, which is a key Sustainable Development Goal, will not be realized.^29^ Market women provide fresh food, fruits, and vegetables to Ghanaians but in spite of this, they face significant health challenges in being sedentary and developing poor dietary habits. Since market women play an important role in the Ghanaian economy, this study was set out to assess the prevalence of obesity among the market women and also investigate the contributing factors to the rising trend of obesity among these women. Evidence concerning the imminent threat of nutrition‐related NCDs will help in making informed decisions on interventions, strategies and policies to curb the disease, spearheaded by relevant stakeholders.

## 2 METHODS

### 2.1 Study design

This was a market‐based cross‐sectional study that involved quantitative methods of data collection. The data was collected from February to May 2020.

### 2.2 Study area

This study was conducted at the Kotokuraba and Abura markets. These are major markets located in the Cape Coast Metropolis and regional capital of the Central Region of Ghana.^12^ These markets serve many communities in the Central Region, providing fresh foods, vegetables, fruits, meat products, fish products, and so forth to residents and travelers in and around Cape Coast.

### 2.3 Study population

The population targeted for the study were market women who conduct their business activities within these markets. The age limit for the respondents was from 18 years upwards, who have been in the market for at least 1 year. The respondents also must have a shed, shop or a permanent place in the market to be recruited and had no known medical condition(s).

### 2.4 Market site observation

The market sites were visited over a period of 4 weeks in January 2020 at different times; at early dawn, morning, midday, evening, and late evening. This was to acquaint ourselves with the time of reporting of the women at the market and time of departure. Their work environment was also noted as well as the foods that were sold in and around the market.

### 2.5 Collection of data

Participants' sociodemographic characteristic information, anthropometry; waist‐to‐hip ratio (WHR), total body fat percentage, visceral fat percentage, height, weight, and 24 h dietary history were collected using semistructured questionnaires. The questionnaire was pretested on 10% sample size in a minimarket near the study site before the actual data collection commenced. This helped to ensure reasonable power in the reliability and validity of the questionnaire. To ensure that quality data was collected, trained interviewers administered the questionnaire to the respondents. Again, the questionnaire was interpreted in the local language where necessary for easy comprehension.

### 2.6 Measurements of height

The height of market women was determined using a portable scale, Seca stadiometer (Seca) calibrated to the nearest 0.1 cm by measuring from the crown of their head to the heel of their feet.

### 2.7 Measurements of weight

Electronic bathroom weighing scales were used to measure weight (Seca Personen). Measurements were taken twice to the nearest 0.1 kg and the average was calculated.

### 2.8 Hip and waist circumference (HC and WC)

The HC and WC were measured using an inextensible tape measure and recorded to the closest 0.1 cm.

### 2.9 Body composition analysis

A transportable and handy device, the Omron body fat analyzer (Omron Healthcare Inc.), which transmits an imperceptible weak electrical current comprising 50 kHz and 500 mA via the body to measure the quantity of fat tissue was used.

### 2.10 DD assessment (24 h recall)

Trained personnel administered validated DD questionnaire by interviewing respondents face‐to‐face. Information regarding respondents' foods eaten and drank for the previous 24 h period were recalled and recorded. Information on portion sizes, methods of cooking were obtained using standard handy measures to determine precise food intake. The recommended 10 food groups according to WHO and the works by Ansari et al.^30^ for defining minimum DD indicator was used in assessing DDS. They were (i) grains, tubers, and roots (ii) pulse (beans, peas, lentils, and so forth), (iii) dairy and dairy products, (iv) flesh foods (meats, fish, and poultry), (v) eggs, (vi) vitamin A‐rich vegetables and fruits, (vii) other fruits, (viii) nuts, seeds, (ix) dark green and leafy vegetables, (x) other vegetables. The DDS is between 0 and 10, where 0 was for food groups that were not consumed and 1 for food groups consumed. The proportion who consumed foods from a minimum of five out of 10 food groups in a 24 h time pace was defined by WHO as having achieved the minimum DD.

### 2.11 Statistical analysis

Data was analyzed using IBM SPSS Statistics, version 21. Descriptive statistics were performed for participants' demography, frequency of daily eating, anthropometric characteristics, and dietary consumption characteristics. All descriptive statistics were expressed as percentage relative to total respondents DDS was presented as respondents scoring between <5 and ≥5. Knowledge in nutrition was presented as respondents who knew food groups and their role in human health. The appropriate cut‐offs were determined for body composition parameters.^31^, ^32^, ^33^ Visceral fat of respondents was group into three; normal (1%−9%), high (10%−14%), and very high (15%−30%). Body fat was categorized into good (16%−23%), acceptable (24%−30%), overweight (31%−36%), and obese (≥37%). BMI was grouped into underweight (<18.5 kg/m^2^), normal (18.5−24.9 kg/m^2^), overweight (25.0−29.9 kg/m^2^), and obese (≥30 kg/m^2^). WHR was obtained by dividing the WC by the HC. This was grouped into low risk (<0.81), moderate risk (0.81−0.85), and high risk (>0.85). The food groups consumed among the 10 food groups were expressed as a percentage. Binary logistic regression analysis was performed for relationship between NK, frequency of daily meals, time of breakfast, lunch, supper, and DD on body composition parameters at a 95% confidence interval (CI) and statistical significance of *p* < 0.05.

### 2.12 Sample size estimation and sampling technique

The minimum sample size estimated for the study was 377. This was determined using the formula by Fisher^34^ to obtain the minimum sample size, and based on the assumed prevalence of 43% of obesity in Ghana^1^ at a confidence interval of 95%; *N* = (*Z* ^2^ × *p* [1−*p*]/*e* ^2^). Specifically, *N* was the minimum sample size estimated; *z* was the point of the standard normal distribution curve which was set at 1.96 (95% CI); *p* was the assumed prevalence rate; *e* was the desired level of precision (the margin of error [0.05]). A total of 402 participants met the inclusion criteria and gave their consent. A simple random technique was employed to recruit the study participants. The number and location of shops, shed, and traders sitting under umbrellas at allocated places were noted. Interviewers randomly moved among these traders to recruit them.

### 2.13 Ethical approval

The Committee on Human Research, Publication and Ethics of the Kwame Nkrumah University of Science and Technology, provided ethical clearance (CHRPE/AP/005/20) for the study. Permission was also granted by appropriate gatekeepers (i.e., The Cape Coast Metropolitan Assembly and the queen mothers of the two market centers) to approach participants for data collection. Informed consent was sought from each participating market woman before being enrolled into the study.

## 3 RESULTS

From Table 1, out of the 402 participants, 26.62% had senior high school education, 9.7% had primary education, and 22.89% had no formal education. Most of the market traders were young as about 51% were aged 20–39 years. About 75% had no knowledge in nutrition and about 60% ate thrice or more daily. Almost half of the traders consume breakfast after 8 a.m., about 62% consume lunch between 1 p.m. and 3 p.m. whiles about 82% take supper from 7 p.m. Majority of the women (91.04%) had a DDS <5. 75.62% of respondents had normal range of visceral fat and 22.89% had high visceral fat. About 65% had high body fat content to be described as obese. The prevalence of obesity was 39.30% and overweight was 31.84%. Majority of the market women consumed starchy staples (99.75%), meat, poultry, and fish (91.29%) and other vegetables (92.79%). Few consumed nuts and seeds (6.0%) and other fruits (2.00%)

**Table 1:** Sociodemographic, anthropometric, and dietary characteristics of study participants.

Variable	Percentage (%)
Market name	
Abura	39.30 (158/402)
Kotokuraba	60.70 (244/402)
Education	
No formal education	22.89 (92/402)
Primary	9.70 (39/402)
Junior high school	23.88 (96/402)
Senior high school	26.62 (107/402)
Tertiary	16.92 (68/402)
Age	
<20	7.71 (31/402)
20−29	32.34 (130/402)
30−39	18.91 (76/402)
40−49	18.16 (73/402)
50−59	14.93 (60/402)
60+	7.96 (32/402)
No of children	
No child	32.34 (130/402)
1−2	26.12 (105/402)
3−4	24.38 (98/402)
5+	17.16 (69/402)
Knowledge in nutrition	
No	74.63 (300/402)
Yes	25.37 (102/402)
Frequency of daily meals	
Once	1.74 (7/402)
Twice	37.56 (151/402)
Thrice	56.97 (229/402)
>Thrice	3.73 (15/402)
Breakfast time	
Before 7 a.m.	19.90 (80/402)
Between 7 & 8 a.m.	31.84 (128/402
After 8 a.m.	48.23 (194/402
Lunch time	
Between 12 & 1 p.m.	31.34 (126/402)
Between 1 & 2 p.m.	37.56 (151/402)
Between 2 & 3 p.m.	25.12 (101/402)
After 3 p.m.	5.97 (24/402)
Supper time	
Before 7 p.m.	18.16 (73/402)
Between 7 and 9 p.m.	72.64 (292/402)
After 9 p.m.	9.20 (37/402)
Dietary diversity	
<5	91.04 (366/402)
≥5	8.96 (36/402)
Visceral fat	
Normal (1%−9%)	75.62 (304/402)
High (10%−14%)	22.89 (92/402)
Very high (15%−30%)	1.49 (6/402)
Body fat	
Good (16%−23%)	3.23 (13/402)
Acceptable (24%−30%)	13.18 (53/402)
Overweight (31%−36%)	18.41 (74/402)
Obese (≥37)	65.17 (262/402)
Body mass index	
Underweight/chronic energy deficiency (<18.5 kg/m^2^)	1.24 (5/402)
Healthy (18.5−24.9 kg/m^2^)	27.61 (111/402)
Overweight (25.0−29.9 kg/m^2^)	31.84 (128/402)
Obese (≥30 kg/m^2^)	39.30 (158/402)
Waist‐to‐hip ratio	
Low risk obesity/oveweight (<0.81)	48.00 (193/402)
Moderate risk (0.81−0.85)	16.17 (65/402)
High risk (>0.85)	35.82 (144/402)
Food group	
Grains, roots, and tubers	99.75 (401/402)
Pulse (beans, peas, lentils)	11.69 (47/402)
Nuts, seeds	6.00 (24/402)
Dairy and dairy products	34.08 (137/402)
Flesh foods (meat, poultry, fish)	91.29 (367/402)
Eggs	15.92 (64/402)
Dark green and leafy vegetables	15.42 (62/402)
Vitamin A‐rich fruits and vegetables	18.91 (76/402)
Other vegetables	92.79 (373/402)
Other fruits	2.00 (8/402)

Table 2 describes the association between NK, frequency of daily meals, time of breakfast, lunch, supper, and DD on body composition parameters. There was significant association between NK and body fat (OR = 0.45; 95% CI = 0.26−0.78, *p* = 0.004) and BMI (OR = 0.40, 95% CI = 0.28−0.71, *p* = 0.001). Frequency of daily meals, breakfast time, lunch time, and supper time had no association with body composition parameters. DD was associated with WHR (OR = 0.40, 95% CI = 0.17−0.95, *p* = 0.03) but no association with other body composition parameters.

**Table 2:** Association between nutrition knowledge, frequency of daily meals, and time of breakfast, lunch, supper, and dietary diversity on body composition parameters.

Variable	*p* Value	Odds ratio (95% confidence interval)
Nutrition knowledge		
Visceral fat	0.008	0.45 (0.25–0.82)
Body fat	0.004	0.45 (0.26–0.78)
Body mass index (BMI)	0.001	0.40 (0.28–0.71)
Waist‐to‐hip ratio (WHR)	<0.001	0.32 (0.19–0.56)
Frequency of daily meals		
Visceral fat	0.90	1.03 (0.65–1.64)
Body fat	0.99	1.01 (0.59–1.72)
BMI	0.59	1.13 (0.73–1.75)
WHR	0.93	0.98 (0.65–1.49)
Breakfast time		
Visceral fat	0.19	0.70 (0.40–1.20)
Body fat	0.29	0.68 (0.33–1.40)
BMI	0.80	1.07 (0.63–1.83)
WHR	0.86	1.05 (0.63–1.75)
Lunch time		
Visceral fat	0.10	1.49 (0.92–2.40)
Body fat	0.66	1.14 (0.64–2.04)
BMI	0.09	1.52 (0.93–2.47)
WHR	0.62	1.12 (0.72–1.73)
Supper time		
Visceral fat	0.51	0.82 (0.46–1.46)
Body fat	0.48	1.26 (0.66–2.43)
BMI	0.58	1.17 (0.67–2.02)
WHR	0.44	0.82 (0.48–1.37)
Dietary diversity		
Visceral fat	0.75	0.88 (0.39–1.99)
Body fat	0.61	0.80 (0.33–1.90)
BMI	0.81	0.91 (0.43–1.93)
WHR	0.03	0.40 (0.17–0.95)

## 4 DISCUSSION

This study comprised 402 market women from two market centers, Abura and Kotokuraba markets, in Cape Coast. The minimum age was 18 years old and respondents were randomly sampled from the market. Knowledge in nutrition^35^ defines awareness of practices and concepts about health and nutrition. This include knowledge of food groups, food as major sources of nutrients and optimal food consumption. From the results, about 75% had no knowledge in nutrition. This is unexpected because about 67% had education spanning from Junior High School to tertiary level. This result is however similar to the findings of Husain et al.^36^ who observed poor NK among prospective teachers studying in the College of Basic Education in Kuwait. Provision of nutrition education for the market traders may help improve their level of knowledge as published by Elmas and Arslan.^37^

It was also discovered that most women (56.97%) ate thrice a day, taking breakfast after 8 a.m. (48.23%) and supper from 7 p.m. (81.84%). The daily routine of the market traders; arriving about 4 a.m. at the market to serve customers and leaving the market at about 7 p.m. to their homes, significantly affect their meal timing. Most market woman are unable to cook in the morning before leaving their houses to the market. Their return to home late at night, may account for the late time of eating supper. Having an irregular meal times has been demonstrated to be associated with elevated risk of metabolic syndrome.^38^

From the study of Kahleova et al.^39^ among seventh‐day Adventist in North America, eating less frequently is an important factor in reducing BMI and eating more than twice daily increases respondents BMI. Paoli et al.^23^ published that regular meal timing and a reduced daily meal frequency may improve human health. Contrary, Speechly et al.^24^ recommended frequently eating (snacking) as a strategy for losing weight. This has been presumed to decrease hunger, energy intake, and body weight. Further studies are needed to confirm the right association

DD measures how varied a person's diet is, in respect of diverse nutrients consumption. Diverse diet is important in the growth, body function, and body composition.^26^ About 91% of the market traders had DDS of <5. This is very worrying because DDS is an indicator of macro and micro nutrients sufficiency, which is required for optimal growth and good health.^27^ From Madlala et al.,^40^ 70.4% of adult respondents in South Africa had DDS < 5. Li et al.,^41^ reported 15% for DDS < 5 for preschoolers in rural China. Differences may result from age groups of respondents. Higher DD and improved lifestyle have been shown to reduce obesity and other cardiometabolic risk in several studies. Chalermsri et al.^28^ reported higher DD was associated with a lower risk of cerebrovascular disease among Thai older people. Again, Gholizadeh et al.^42^ reported lower DDS was associated with high probability of metabolic syndrome and they all recommend interventions increasing the DDS with good fruits, fresh vegetables, and whole grains because they have positive influence on obesity.

This study also presented obesity prevalence of 39.30% and overweight 31.84%. Similar results from Awosan et al.^10^ presented a prevalence of overweight as 28.9%, and obesity 28.1%. Again results from Ofori‐Asenso et al.^1^ presented overweight/obesity at 43% and Asosega et al.^8^ reported the general prevalence of overweight/obesity among women of reproductive age in Ghana as 35.4%. This high prevalence may be brought about because the market women consume high amounts of starchy staples and other energy dense foods. These women are at a greater risk of diabetes, ischemic heart diseases, and certain cancers^7^ and they are likely to be poor or remain poor because of health cost and a decreased life expectancy ^5^

There was significant association between knowledge in nutrition and all body composition parameters; body fat (*p* = 0.004), BMI (*p* = 0.001), and WHR (*p* < 0.001), and visceral fat (*p* = 0.008). Brien and Davies^43^ presented no significant correlation between levels of knowledge and BMI, Yahia et al.^44^ presented a negative correlation between NK and fat and cholesterol intake. It is believed that delivery and improvement of nutrition education could improve nutrition status,^20^, ^45^ and stakeholders must enact policies to ensure a healthy population.

From the results, there was no significant relationship between DDS and body composition parameters, except WHR (*p* = 0.03). Other publications presented no significant relationship between DDS and body composition parameters. For example, Samukelisiwe et al.^40^ published no association between DDS and BMI and WHR. Salehi‐Abargouei et al.^25^ meta‐analysis showed no significant relationship between DD and BMI. Several publications have also demonstrated different relationship between DDS and BMI, body fat, and visceral fat. In Azadbakhet et al.^27^ research conducted on Iranian adults, a negative association was observed between DDS, obesity, and abdominal obesity. Again, two other studies undertaken by Kant et al.^46^, ^47^ presented an inverse correlation between DDS and BMI. These inconsistent findings may be as a result of different serving cut‐off points which were used to measure the DD. Further prospective investigations are needed to confirm the association.

## 5 LIMITATIONS

There are some limitations to the study. Because this study is a cross‐sectional design, we are careful in making conclusions about the cause and effect. A larger random sample size, coupled with an accurate dietary assessment method such as replicate quantitative 24 h recalls would improve its strength to establish reliable associations. Notwithstanding, this study has been able to provide association between DD, body fat, and visceral fat. Another limitation to this study was the small sample size. Nevertheless, the random sampling and similar characteristics of respondents to the general populace of the Central Region allows for some measure of generalization of findings in the current study to women in the region. As a result, carrying out this study even with a small sample will bring out new ideas in the field of DDS, NK, attitudes, and body composition.

## 6 CONCLUSION

The prevalence of obesity and overweight was high among the market women and they had poor DD and poor NK. More attention should paid to our market centers where these women are mostly concentrated. Effective nutrition education and a change in their attitude will ultimately result in improved DD and nutrition status. Further prospective investigations could examine association between DD and BMI in an in‐depth manner.

## AUTHOR CONTRIBUTIONS

**Charles Apprey**: Conceptualization; investigation; methodology; resources; supervision; writing—review and editing. **Martina Mensah**: Conceptualization; formal analysis; methodology; resources. **Desmond Agyarko**: Conceptualization; formal analysis; methodology; resources; writing—original draft; writing—review and editing. **Yaa Asantewaa K. Klu**: Conceptualization; methodology; resources; supervision; writing—review and editing. **Samuel Acquah**: Methodology; resources; supervision; writing—review and editing. All authors have read and approved the final version of the manuscript.

## CONFLICT OF INTEREST STATEMENT

The authors declare no conflict of interest.

## TRANSPARENCY STATEMENT

The lead author Charles Apprey affirms that this manuscript is an honest, accurate, and transparent account of the study being reported; that no important aspects of the study have been omitted; and that any discrepancies from the study as planned (and, if relevant, registered) have been explained.

## Data Availability

The data and materials are available in the corresponding author's institution and will be made available upon formal request. Corresponding author (Charles Apprey) had full access to all of the data in this study and takes complete responsibility for the integrity of the data and the accuracy of the data analysis.
